# Development and validation of experimental induction tasks for worry and rumination: A comparison of scripted and personalized approaches

**DOI:** 10.21203/rs.3.rs-5139533/v1

**Published:** 2024-09-26

**Authors:** Hanjoo Kim, Michelle Newman

**Affiliations:** University of Michigan; Pennsylvania State University

**Keywords:** repetitive negative thought, worry, rumination, induction, experiment

## Abstract

Worry and rumination are two forms of repetitive negative thoughts. Prior studies have identified similarities and differences between these two states. For a more comprehensive understanding of these thought processes, researchers need reliable methods to induce them experimentally. Traditionally, researchers have used either scripted or personalized approaches to trigger worry and rumination, but it remains unclear which method is more effective. Additionally, the potential impact of preexisting disorders like generalized anxiety disorder (GAD) or major depression (MDD) on these inductions is not well understood. This study aimed to compare scripted and personalized induction methods to identify which was more effective for eliciting worry and rumination, while also considering the potential influence of generalized anxiety and depression. Participants (*N* = 355) included individuals with GAD, depression, or healthy controls. They were randomly assigned to either scripted or personalized induction tasks designed to induce worry or rumination. Findings revealed that personalized induction methods were consistently more effective than scripted methods for inducing both worry and rumination, regardless of participants’ group characteristics. In addition to the general underperformance of scripted induction methods, the scripted rumination induction was notably less successful, failing to induce rumination to a greater extent than the scripted worry induction. Given these findings, personalized approaches are recommended for experimental studies that aim to compare worry and rumination. Limitations of the study and implications for future research are also discussed.

## Introduction

Worry and rumination are two types of repetitive negative thoughts that lead to emotional distress. Worry refers to excessive and uncontrollable thoughts about potential threats (Borkovec et al., 1983). Rumination involves perseverative thinking about the causes and consequences of one’s problems and associated feelings (Nolen-Hoeksema, 1991). Unlike worry, which involves persistent thoughts on future events, rumination often involves past failures (Kingston et al., 2014; Nolen-Hoeksema et al., 2008). Worry is considered a defining feature of generalized anxiety disorder (GAD; Newman et al., 2013), whereas rumination has been recognized as a key characteristic of major depressive disorder (MDD; Nolen-Hoeksema et al., 2008).

Despite conceptual differences, there is considerable overlap between worry and rumination. They often occur interchangeably in individuals with GAD and MDD (Erickson et al., 2020; Watkins et al., 2005). Measures of worry and rumination were highly correlated in both clinical and non-clinical samples (Segerstrom et al., 2000; Szkodny & Newman, 2019). Some studies suggested that they shared common latent structures (Segerstrom et al., 2000; Topper et al., 2014). Additionally, worry and rumination were found not only in GAD and MDD but also in bipolar spectrum disorders, posttraumatic stress disorder, and social anxiety disorder (Hearn et al., 2017; Mellings & Alden, 2000; Moulds et al., 2020; Sarisoy et al., 2014; Silveira Jr & Kauer-Sant’Anna, 2015; Tull et al., 2011). Thus, worry and rumination may represent transdiagnostic mechanisms for emotion dysregulation, cutting across various affective disorders.

There is also a convergence between worry and rumination. Worry aligned with a cognitive bias away from threat, whereas rumination showed bias toward loss and failure (Hur et al., 2019). Worry was associated with lower heart rate variability, whereas rumination was less strongly linked to this measure (Aldao et al., 2013). Worry was often related to appraisal of low coping effectiveness, whereas rumination led to disengagement from problem-solving (Hong, 2007). These findings underscore the need for more research to clarify shared and unique aspects of worry and rumination.

Understanding the intricacies of worry and rumination is key to creating more targeted interventions. Laboratory studies offer controlled environments, allowing researchers to isolate specific variables and examine their causal impact on these cognitive patterns. Researchers often use methods to induce worry and rumination to compare their effects on various outcomes. However, a major challenge in comparing worry and rumination has been the inconsistency of induction methods, which hampers meaningful side-by-side analysis.

Worry induction typically relies on “personalized” scenarios, where participants are prompted to think about potential worrisome events in their lives. This approach, developed by Borkovec and Inz (1990) is widely used in experimental studies on worry (e.g., Behar et al., 2005; Fisher & Newman, 2013; Llera & Newman, 2010, 2014, 2020; Oathes et al., 2008; Zainal & Newman, 2018). At the same time, traditional rumination inductions are typically “scripted,” involving participants reading statements prepared by researchers. This method was introduced by Nolen-Hoeksema and Morrow (1993) and is commonly used in studies on rumination (e.g., LeMoult & Joormann, 2014; Watkins & Brown, 2002; Whitmer & Gotlib, 2012).

Experimental studies comparing worry and rumination generally used one of these two approaches: some adopted personalized inductions to elicit worry and rumination (e.g., Jamil & Llera, 2021; Kim & Newman, 2022; Kim & Newman, 2023; McLaughlin et al., 2007), whereas others relied on scripted inductions (e.g., Capobianco et al., 2018; Lewis et al., 2019). Yet, the rationale for selecting one method over the other has not been thoroughly examined. There is no consensus on which is more effective. Additionally, it remains unclear if observed differences in worry and rumination are influenced by pre-existing group characteristics, such as anxiety or depression. To address these uncertainties, this study aimed to compare the effectiveness of personalized and scripted induction methods, while also considering the potential impact of group characteristics.

We sought to answer the following questions: 1. Which induction method, between scripted and personalized, was more effective at inducing the targeted response? 2. Did the induction methods produce stronger responses when they matched the intended emotion? 3. To what extent did group characteristics influence the effects of each induction method?

## Method

### Participants

Participants were recruited from a university subject pool, with a total of 356 individuals. Due to a computer error during the experiment, one participant’s data was excluded, resulting in a final sample of 355, including 118 with GAD (and low depression), 113 with depression (and low GAD), and 124 healthy controls (HC). Participants were assigned to the GAD group if they met the full diagnostic criteria on the Generalized Anxiety Disorder Questionnaire-IV (GAD-Q-IV; Newman et al., 2002) and scored 13 or less on the Beck Depression Inventory-II (BDI-II; Beck, Steer, & Brown, 1996). They were included in the depression group if they scored 20 or above on the BDI-II and did not meet the diagnostic criteria on the GAD-Q-IV. Participants were categorized as HC if they neither met the diagnostic criteria on the GAD-Q-IV nor scored 14 or above on the BDI-II. Participants were randomly assigned to scripted (either scripted worry or scripted rumination) or personalized inductions (either personalized worry or personalized rumination), with stratification by group (GAD, depression, and HC)[1]. The sample was predominantly female (282 women, 79.4% vs. 73 men, 20.6%). The racial composition included 267 White (75.2%), 37 Asian (10.4%), 22 Hispanic (6.2%), 18 Black or African-American (5.1%), and 11 from other racial backgrounds (3.1%). The mean age was 18.23 (*SD* = 2.83). The average score for the GAD-Q-IV (using continuous scoring) was 4.88 (*SD* = 3.88), whereas the average BDI-II score was 12.35 (*SD* = 10.42). Further details on participant characteristics can be found in Table 1.

### Procedure

This study was approved by the Institutional Review Board at the authors’ affiliated institution. Prior to the experiment, participants provided written consent, and the concepts of worry and rumination were explained to them. Following Borkovec et al. (1983) and Nolen-Hoeksema (1991), worry was defined as “a chain of uncontrollable thoughts and images about things that might happen in the future,” whereas rumination was described as “passively and repetitively thinking about possible causes, implications, and consequences of stressful events and negative feelings as opposed to its solutions.” The experiment began with a 5-minute resting baseline to help participants acclimate to the setting. They then completed the assigned perseverative thought induction task. In the two personalized induction groups, they engaged either with their most worrisome or ruminative topics as vividly as possible for two minutes. In the two scripted induction groups, participants read a list of either worrisome or ruminative topics for eight minutes at their own pace. After the baseline and induction phase, participants rated their levels of worry and rumination. They received research credits for participating. All procedures were implemented using E-Prime 2.0 (Psychology Software Tools inc., 2002).

### Instruments

#### Generalized Anxiety Disorder Questionnaire-IV (Newman et al., 2002)

The GAD-Q-IV is a nine-item self-report measure assessing GAD symptoms following diagnostic criteria outlined in the Diagnostic and Statistical Manual of Mental Disorders (American Psychiatric Association, 2013). Diagnoses can be established either through a dimensional cutoff or by determining if individuals meet full diagnostic criteria. In this study, we opted for criterion scoring based on a prior study (Moore et al., 2014) demonstrating that it provided higher sensitivity (89%) and specificity (82%) than dimensional scoring. The internal consistency of the current sample was great (Cronbach’s α = .82)[2].

### Beck Depression Inventory-II (Beck, Steer, & Brown, )

The BDI-II is a 21-item self-report questionnaire that assesses major depressive disorder symptoms. It demonstrated good convergent and discriminant validity (Beck, Steer, & Brown, 1996; Steer et al., 1999) and high retest reliability (Beck et al., 1988). A cutoff score of 18 has high sensitivity (94%) and specificity (92%) (Arnau et al., 2001). We used a more rigorous cutoff – 20 or above for moderate to severe depression, and 13 or less for minimal depression, as suggested by the original study (Beck, Steer, Ball, et al., 1996) - to screen participants. Internal consistency in our sample was strong (Cronbach’s α = .92).

### Subjective Emotion Scales

During both the resting baseline and induction phases, participants were asked to rate their levels of worry and rumination using a 9-point Likert scale. The scale ranged from 0, indicating “not at all”, to 8, indicating “extremely”.

### Scripted Induction Methods

For script-based induction of worry, we presented items from the Worry Domains Questionnaire (WDQ; Tallis et al., 1994). The WDQ comprises 25 items selected for their high intensity and frequency from the General Worry Questionnaire (GWQ; Tallis et al., 1992). The WDQ covers a wide range of worry-provoking topics, such as relationships, an uncertain future, work-related concerns, and financial issues. Following previous studies (e.g., Freeman et al., 2013; Ikani et al., 2022), we instructed participants to read through the worry-inducing topics at their own pace for eight minutes.

For the scripted rumination induction, we used the method developed by Nolen-Hoeksema and Morrow (1993), consisting of 45 statements that encourage reflection on oneself, along with the causes and consequences of current feelings and situations. Participants read these statements at their own pace for eight minutes. Additional information is available in Method S1.

### Personalized Induction Methods

For personalized worry and rumination inductions, we adapted Borkovec and Inz (1990)’s induction method. Before the experiment, participants wrote five scenarios that triggered the most intense worry or rumination for them. They then practiced thinking about each scenario for one minute and rated their level of worry and rumination on a 9-point Likert scale from 0 (not at all) to 8 (extremely). To ensure the scenarios were suitable for the experiment, we pre-screened them using a set of standards. To qualify for emotion manipulation, the target emotion had to score above 4 on the scale and at least 3 points higher than the non-target emotions. Additionally, the temporal orientation had to align with the induction type: for worry, it needed to be future-oriented (above 4), and for rumination, past-oriented (below 4). During the induction period, participants engaged with their most intense worry or rumination scenario for two minutes. Further details are provided in Method S2.

### Analytic plan

All analyses were conducted using RStudio version 2023.12.0 + 369 (RStudio Team, 2023). We computed descriptive statistics for demographic characteristics and GAD-Q-IV and BDI-II scores. To assess differences across groups and experimental conditions, we used ANOVA and chi-square tests, followed by pairwise comparisons when results were significant. To compare different induction methods (scripted vs. personalized) and types (worry induction vs. rumination induction), we used random-intercept linear mixed models. These models included group, induction (either method or type), time (resting baseline vs. induction phase), their interactions, and demographic covariates (age, gender, and race). When a significant two-way interaction between induction and time, or a three-way interaction among induction, time, and group was found, we conducted simple slope analyses to examine the significance of the slopes and the differences between them.

## Results

### Descriptive statistics

As expected, the GAD group had higher GAD-Q-IV scores than both the depression and HC groups, whereas the depression group had higher scores than HC (with all *p*-values < .001). The depression group had higher BDI-II scores than the GAD group and HC, whereas the GAD group scored higher than HC. Age did not vary significantly across groups. Similarly, there were no significant differences in gender distribution or racial identity. Group difference statistics are detailed in Table 1.

We also tested for differences across experimental conditions. Neither age, *F*(3, 170.75) = 2.22, *p* = .087, *η*^*2*^ = .04, nor gender, χ^2^(3) = 1.16, *p* = .762, *V* = .06, nor racial identity, χ^2^(12) = 15.42, *p* = .219, *V* = .12 showed significant differences across conditions. There were also no significant differences in continuous GAD-Q-IV scores, *F*(3, 194.85) = 1.61, *p* = .189, *η*^*2*^ = .02, and BDI-II scores, *F*(3, 194.36) = .37, *p* = .774, *η*^*2*^ = .01. Thus, participant assignment to experimental conditions was balanced.

### Comparison of scripted vs. personalized worry induction method in the induction of worry

**Figure 1:**
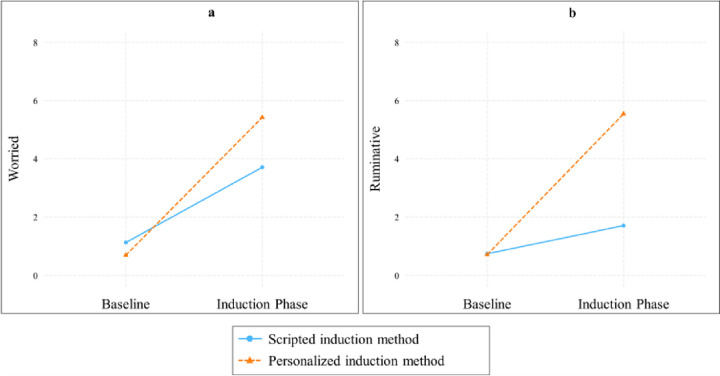
Interaction plot illustrating the effects of different induction methods (scripted vs. personalized) on target emotions *Note*. Fig. 1a depicts the impact of scripted vs. personalized worry inductions on levels of worry, whereas Fig. 1b illustrates the effects of scripted vs. personalized rumination inductions on levels of rumination.

For the worry induction, there was a significant two-way interaction between induction method and time, *F*(1, 176.47) = 22.06, *p* < .001, *η*^*2*^ = .11. Both the scripted, *B* = 2.58, *SE* = .40, *p* < .001, *d* = 1.40 and personalized worry induction, *B* = 4.73, *SE* = .29, *df* = 87.00, *d* = 3.44 increased worry from the resting baseline. The comparison of slopes showed that the personalized worry induction increased worry more than the scripted worry induction, *t*(175) = −4.34, *p* < .001, *d* = .66. Figure 1a illustrates the two-way interaction and Table 2 shows the means and standard deviations for worry scores at both the resting baseline and the induction phase. The three-way interaction between worry induction method, time, and group was not significant, *F*(1, 176.47) = 2.48, *p* = .087, *η*^*2*^ = .03.

### Comparison of scripted vs. personalized rumination induction method in the induction of rumination

During the rumination induction, a significant two-way interaction between the induction method and time was observed, *F*(1, 174.20) = 73.02, *p* < .001, *η*^*2*^ = .30. Both scripted, *B* = .97, *SE* = .34, *p* = .01, *d* = .62 and personalized rumination inductions, *B* = 4.83, *SE* = .46, *p* < .001, *d* = 2.25 led to significant increases in rumination. However, the personalized rumination induction increased rumination more than the scripted rumination induction, *t*(172) = −6.75, *p* < .001, *d* = 1.03. The two-way interaction effect is illustrated in Fig. 1b, and the means and standard deviations for rumination scores at the resting baseline and during the induction phase are provided in Table 2. The three-way interaction between induction method, time, and group was not significant, *F*(1, 174.20) = 2.37, *p* = .096, *η*^*2*^ = .03.

### Comparison of scripted worry vs. scripted rumination induction in the induction of worry

**Figure 2:**
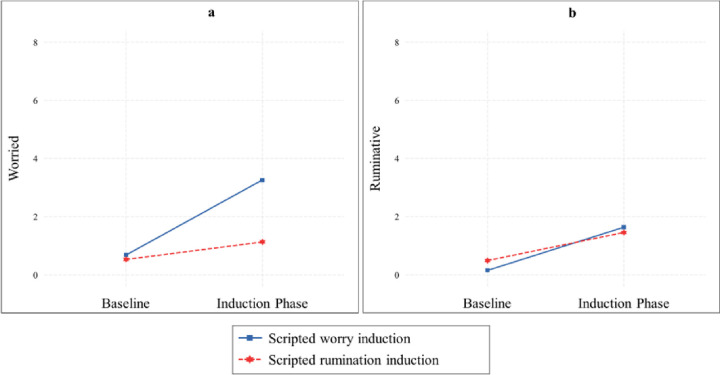
Interaction plot illustrating the effects of different scripted induction conditions (scripted worry vs. scripted rumination induction) on levels of worry and rumination *Note*. Fig. 2a depicts the impact of scripted worry vs. scripted rumination induction on levels of worry, whereas Fig. 2b illustrates the effects of scripted worry vs. scripted rumination induction on levels of rumination.

We observed a significant two-way interaction between scripted induction type (worry vs. rumination) and time on worry, *F*(1, 169.00) = 40.44, *p* < .001, *η*^*2*^ = .19. Both the scripted worry induction, *B* = 2.58, *SE* = .40, *p* < .001, *d* = 1.40 and scripted rumination induction, *B* = .60, *SE* = .30, *p* = .047, *d* = .44 led to significant increases in worry. However, the scripted worry induction increased worry more than did the scripted rumination induction, *t*(171) = 3.99, *p* < .001, *d* = .61. Figure 2a illustrates the two-way interaction effect and Table 2 demonstrates the means and standard deviations for worry scores at both resting baseline and the induction phase. The three-way interaction between induction type, time, and group was not observed, *F*(1, 169.00) = 2.78, *p* = .065, *η*^*2*^ = .03.

### Comparison of scripted worry vs. scripted rumination induction in the induction of rumination

The two-way interaction between scripted induction type and time indicated no significant difference between the worry script and rumination script on increased rumination, *F*(1, 169.00) = 2.00, *p* = .159, *η*^*2*^ = .01. A significant increase in rumination was observed in both the scripted worry induction, *B* = 1.48, *SE* = .36, *p* < .001, *d* = .88, and the scripted rumination induction, *B* = .97, *SE* = .34, *p* = .006, *d* = .62. Figure 2b illustrates the non-significant two-way interaction between induction type and time and Table 2 displays the means and standard deviations for rumination scores at resting baseline and during the induction phase. Additionally, the three-way interaction between induction type, time, and group was not significant, *F*(1, 169.00) = 1.29, *p* = .277, *η*^*2*^ = .02.

### Comparison of personalized worry vs. personalized rumination induction in the induction of worry

**Figure 3:**
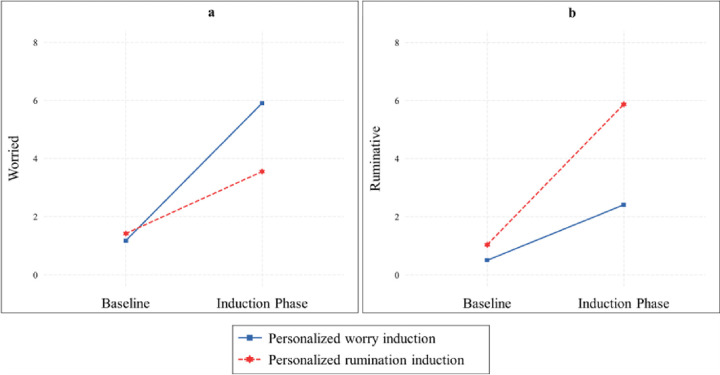
Interaction plot illustrating the effects of different personalized induction conditions (personalized worry vs. personalized rumination induction) on levels of worry and rumination *Note*. Fig. 3a depicts the impact of personalized worry vs. personalized rumination induction on levels of worry, whereas Fig. 3b illustrates the effects of personalized worry vs. personalized rumination induction on levels of rumination.

During the personalized induction, we observed a significant two-way interaction between induction type (worry vs. rumination) and time, *F*(1, 174.00) = 63.93, *p* < .001, *η*^*2*^ = .27, on increased worry. Both the personalized worry induction, *B* = 4.73, *SE* = .29, *p* < .001, *d* = 3.44 and the personalized rumination induction, *B* = 2.13, *SE* = .45, *p* < .001, *d* = 1.01 significantly increased worry from the resting baseline. However, the comparison of slopes indicated that the personalized worry induction increased worry more than the personalized rumination induction, *t*(176) = 4.79, *p* < .001, *d* = .72. The two-way interaction between induction type and time is illustrated in Fig. 3a, and means and standard deviations for worry scores at both resting baseline and during the induction phase are provided in Table 2. In this model, the three-way interaction between induction type, time, and group was not significant, *F*(1, 174.00) = .53, *p* = .587, *η*^*2*^ = .01.

### Comparison of personalized worry vs. personalized rumination induction in the induction of rumination

In the induction of rumination, there was a significant two-way interaction between induction type and time, *F*(1, 174.00) = 35.75, *p* < .001, *η*^*2*^ = .17. Both the personalized worry induction, *B* = 1.91, *SE* = .38, *p* < .001, *d* = 1.07 and personalized rumination induction, *B* = 4.83, *SE* = .46, *p* < .001, *d* = 2.25 significantly increased rumination from the resting baseline. However, the slope comparison showed that the personalized rumination induction increased rumination more than the personalized worry induction, *t*(176) = −4.89, *p* < .001, *d* = .74. The two-way interaction between induction type and time is illustrated in Fig. 3b and the means and standard deviations for rumination scores at both the resting baseline and during the induction phase are displayed in Table 2. On the other hand, the three-way interaction between induction type, time, and group was not observed, *F*(1, 174.00) = 1.84, *p* = .161, *η*^*2*^ = .02.

## Discussion

This study compared the effectiveness of personalized and scripted methods for inducing worry and rumination, an area that has not been thoroughly explored. We also examined whether these methods were more effective when aligned with the targeted repetitive thought process and whether their effects varied by diagnostic group. Our findings showed that the personalized method consistently outperformed the scripted method in eliciting the targeted repetitive thought process. These results held true regardless of group characteristics. Furthermore, the personalized induction led to more pronounced effects when the induction type matched the target repetitive thought process. Specifically, worry was induced more by the personalized worry induction than by personalized rumination induction, whereas rumination was evoked more by the personalized rumination induction than by the personalized worry induction, providing support for the validity of the personalized induction methods.

Beyond the overall underperformance of the scripted method, the scripted rumination induction, in particular, presented specific issues. Although the scripted worry induction increased worry more than the scripted rumination induction, the scripted rumination induction did not increase rumination more than did the scripted worry induction. Thus the scripted rumination induction was no more effective at inducing rumination than the scripted worry induction.

This discrepancy may stem from the original objectives behind the development of the scripted rumination induction method. Unlike the worry induction developed by Borkovec and Inz (1990), and designed to elicit worry, Nolen-Hoeksema and Morrow (1993)’s rumination induction was originally designed to test the effects of reflective thought on depressive mood among individuals with depression. As a result, the content of the induction task was intentionally made to represent a more neutral form of mentation rather than explicitly negative themes. Although this approach helps identify thought patterns unique to those with depression, it may explain its reduced efficacy in inducing “depressive rumination,” which involves more overtly negative thinking (Conway et al., 2000; Papageorgiou & Wells, 2004; Robinson & Alloy, 2003).

Experimental studies wherein worry or rumination are induced, are important because they allow researchers to determine the causal impact of these repetitive thought processes. As noted above, worry and rumination have been implicated as transdiagnostic and are potentially causally related to the maintenance of many psychological disorders (Erickson et al., 2020). Multiple experimental studies have been conducted to confirm the similar or different effects of these thought processes on subjective and objective indices of emotion (e.g., Ottaviani et al., 2016). However, the current study suggests that the specific induction methods used may lead to significantly different outcomes. In particular, we highly recommend using the personalized induction method, regardless of the thought process targeted as this method was more effective at inducing the targeted thought process. Future studies might also examine the induction of angry rumination and or obsessive thoughts.

It is important to acknowledge the limitations of this study. Although this study distinguished between GAD and depression to assess their separate effects on worry and rumination inductions, it is not uncommon for these conditions to coexist in the same individual. Including samples with comorbid GAD and depression could reveal whether having both disorders results in greater worry and rumination induction compared to having either condition alone. Furthermore, whereas we applied strict standards for participant screening, this study focused on a college sample, resulting in a relatively homogeneous group in terms of age and education. To enhance the generalizability of our findings, future research should aim to replicate this study with treatment-seeking populations from more diverse backgrounds.

Our study demonstrated that personalized induction methods were more effective and appropriate for inducing worry and depressive rumination. We hope these findings will ease the burden on researchers in choosing a valid induction method and contribute to advancing knowledge and practices to help individuals grapple with repetitive negative thoughts.

## Figures and Tables

**Table 1:** Demographic characteristics

Variable	Total (*N* = 355)	GAD (*n* = 118)	Depression (*n* = 113)	HC (*n* = 124)	Statistics		
Categorical variables					χ^2^	*p*	*V*
Gender, *n* (%)					2.16	.340	.08
Women	282 (79.4)	99 (83.9)	87 (77.0)	96 (77.4)			
Men	73 (20.6)	19 (16.1)	26 (23.0)	28 (22.6)			
Race, *n* (%)					9.08	.336	.11
White	267 (75.2)	93 (75.0)	81 (65.3)	93 (75.0)			
Asian	37 (10.4)	13 (10.5)	13 (10.5)	11 (8.9)			
Hispanic	22 (6.2)	6 (4.8)	9 (7.3)	7 (5.6)			
African-American	18 (5.1)	1 (0.8)	8 (6.5)	9 (7.3)			
Others	11 (3.1)	5 (4.0)	2 (1.6)	4 (3.2)			
Continuous variables					*F*	*P*	*ŋ^2^*
Age, *M (SD)*	18.23 (2.83)	18.30 (3.21)	18.34 (2.85)	18.06 (2.42)	.377	.687	.00
GAD-Q-IV, *M (SD)*	4.88 (3.88)	9.10 (1.58)	4.40 (3.10)	1.30 (1.35)	846.47	<.001	.89
BDI-2, *M (SD)*	12.35 (10.42)	8.92 (2.93)	25.75 (6.19)	3.39 (3.50)	570.8	<.001	.84

*Note.* GAD, Generalized Anxiety Disorder; HC, Healthy Controls; GAD-Q-IV, Generalized Anxiety Disorder Questionnaire-IV-Continuous Score; BDI-2, Beck Depression Inventory-2

**Table 2:** Means and standard deviations for worry and rumination scores from resting baseline to induction phase by induction method (scripted vs. personalized) and induction type (worry induction vs. rumination induction)

Variables	Scripted method				Personalized method			
	Worry induction		Rumination induction		Worry induction		Rumination induction	
	Baseline	Induction	Baseline	Induction	Baseline	Induction	Baseline	Induction
Worry score, *M(SD)*	1.03 (1.50)	3.97 (2.10)	1.07 (1.51)	2.12 (1.94)	.79 (1.26)	5.14 (1.43)	1.26 (1.70)	3.03 (2.11)
Rumination score, *M(SD)*	.90 (1.37)	2.84 (2.28)	1.17 (1.63)	2.69 (2.38)	.70 (1.20)	2.94 (2.33)	1.33 (1.85)	5.72 (1.81)

*Note.* Worry and rumination scores were rated on the 9-point Likert scale ranging from 0 to 8.

## References

[1] Aldao A., Mennin D. S., & McLaughlin K. A. (2013). Differentiating worry and rumination: Evidence from heart rate variability during spontaneous regulation. Cognitive Therapy and Research, 37(3), 613–619. 10.1007/s10608-012-9485-0 25284916 PMC4180405

[2] American Psychiatric Association. (2013). Diagnostic and Statistical Manual of Mental Disorders (DSM-5; 5th ed.). American Psychiatric Association. 10.1176/appi.books.9780890425596

[3] Arnau R. C., Meagher M. W., Norris M. P., & Bramson R. (2001). Psychometric evaluation of the Beck Depression Inventory-II with primary care medical patients. Health Psychology, 20(2), 112–119. 10.1037/0278-6133.20.2.112 11315728

[4] Beck A. T., Steer R. A., Ball R., & Ranieri W. F. (1996). Comparison of Beck Depression Inventories-IA and-II in psychiatric outpatients. Journal of Personality Assessment, 67(3), 588–597. 10.1207/s15327752jpa6703_13 8991972

[5] Beck A. T., Steer R. A., & Brown G. K. (1996). Beck depression inventory-II. APA PsychTests. 10.1037/t00742-000

[6] Beck A. T., Steer R. A., & Garbin M. G. (1988). Psychometric properties of the Beck Depression Inventory: Twenty-five years of evaluation. Clinical Psychology Review, 8(1), 77–100. 10.1016/0272-7358(88)90050-5

[7] Behar E., Zuellig A. R., & Borkovec T. D. (2005). Thought and imaginal activity during worry and trauma recall. Behavior Therapy, 36(2), 157–168. 10.1016/S0005-7894(05)80064-4

[8] Borkovec T. D., & Inz J. (1990). The nature of worry in generalized anxiety disorder: A predominance of thought activity. Behaviour Research and Therapy, 28(2), 153–158. 10.1016/0005-7967(90)90027-G 2183759

[9] Borkovec T. D., Robinson E., Pruzinsky T., & DePree J. A. (1983). Preliminary exploration of worry: Some characteristics and processes. Behaviour Research and Therapy, 21(1), 9–16. 10.1016/0005-7967(83)90121-3 6830571

[10] Capobianco L., Morris J. A., & Wells A. (2018). Worry and rumination: Do they prolong physiological and affective recovery from stress? Anxiety Stress and Coping, 31(3), 291–303. 10.1080/10615806.2018.1438723 29433340

[11] Conway M., Csank P. A., Holm S. L., & Blake C. K. (2000). On assessing individual differences in rumination on sadness. Journal of Personality Assessment, 75(3), 404–425. 10.1207/S15327752JPA7503_04 11117154

[12] Erickson T. M., Newman M. G., & Tingey J. L. (2020). Worry and Rumination. In Abramowitz J. S. & Blakey S. M. (Eds.), Clinical handbook of fear and anxiety: Maintenance processes and treatment mechanisms (pp. 133–151). American Psychological Association. 10.1037/0000150-008

[13] Fisher A. J., & Newman M. G. (2013). Heart rate and autonomic response to stress after experimental induction of worry versus relaxation in healthy, high-worry, and generalized anxiety disorder individuals. Biological Psychology, 93(1), 65–74. 10.1016/j.biopsycho.2013.01.012 23384513

[14] Freeman D., Startup H., Dunn G., Černis E., Wingham G., Pugh K., Cordwell J., & Kingdon D. (2013). The interaction of affective with psychotic processes: a test of the effects of worrying on working memory, jumping to conclusions, and anomalies of experience in patients with persecutory delusions. Journal of psychiatric research, 47(12), 1837–1842. 10.1016/j.jpsychires.2013.06.016 23871449 PMC3905189

[15] Hearn C. S., Donovan C. L., Spence S. H., & March S. (2017). A worrying trend in Social Anxiety: To what degree are worry and its cognitive factors associated with youth Social Anxiety Disorder? Journal of Affective Disorders, 208, 33–40. 10.1016/j.jad.2016.09.052 27744124

[16] Hong R. Y. (2007). Worry and rumination: Differential associations with anxious and depressive symptoms and coping behavior. Behaviour Research and Therapy, 45(2), 277–290. 10.1016/j.brat.2006.03.006 16635479

[17] Hur J., Gaul K., & Berenbaum H. (2019). Different patterns of attention bias in worry and rumination. Cognitive Therapy and Research, 43(4), 713–725. 10.1007/s10608-018-09993-4

[18] Ikani N., Radix A. K., Rinck M., & Becker E. S. (2022). Changing metacognitive appraisal bias in high-worriers through reappraisal training. Cognitive Therapy and Research, 46(4), 852–863. 10.1007/s10608-022-10297-x

[19] Jamil N., & Llera S. J. (2021). A transdiagnostic application of the contrast-avoidance model: The effects of worry and rumination in a personal-failure paradigm. Clinical Psychological Science, 9(5), 836–849. 10.1177/2167702621991797

[20] Kim H., & Newman M. G. (2022). Avoidance of a negative emotional contrast from worry and rumination: An application of the contrast avoidance model. Journal of Behavioral and Cognitive Therapy, 32(1), 33–43. 10.1016/j.jbct.2021.12.007 35693377 PMC9181176

[21] Kim H., & Newman M. G. (2023). Worry and rumination enhance positive emotional contrast based on the framework of the Contrast Avoidance Model. Journal of Anxiety Disorders, 102671. 10.1016/j.janxdis.2023.102671 36681058 PMC10071830

[22] Kingston R. E., Watkins E. R., & Nolen–Hoeksema S. (2014). Investigating functional properties of depressive rumination: Insight and avoidance. Journal of Experimental Psychopathology, 5(3), 244–258. 10.5127/jep.038013

[23] LeMoult J., & Joormann J. (2014). Depressive rumination alters cortisol decline in Major Depressive Disorder. Biological Psychology, 100, 50–55. 10.1016/j.biopsycho.2014.05.001 24835412 PMC4101056

[24] Lewis E. J., Blanco I., Raila H., & Joormann J. (2019). Does repetitive negative thinking affect attention? Differential effects of worry and rumination on attention to emotional stimuli. Emotion, 19(8), 1450–1462. 10.1037/emo0000535 30714778

[25] Llera S. J., & Newman M. G. (2010). Effects of worry on physiological and subjective reactivity to emotional stimuli in generalized anxiety disorder and nonanxious control participants. Emotion, 10(5), 640–650. 10.1037/a0019351 21038947

[26] Llera S. J., & Newman M. G. (2014). Rethinking the role of worry in generalized anxiety disorder: Evidence supporting a model of Emotional Contrast Avoidance. Behavior Therapy, 45(3), 283–299. 10.1016/j.beth.2013.12.011 24680226

[27] Llera S. J., & Newman M. G. (2020). Worry impairs the problem-solving process: Results from an experimental study. Behaviour Research and Therapy, 135, 103759. 10.1016/j.brat.2020.103759 33129156 PMC7703801

[28] McLaughlin K. A., Borkovec T. D., & Sibrava N. J. (2007). The effects of worry and rumination on affect states and cognitive activity [JOUR]. Behavior Therapy, 38(1), 23–38. 10.1016/j.beth.2006.03.003 17292692

[29] Mellings T. M., & Alden L. E. (2000). Cognitive processes in social anxiety: The effects of self-focus, rumination and anticipatory processing. Behaviour Research and Therapy, 38(3), 243–257. 10.1016/S0005-7967(99)00040-6 10665158

[30] Moore M. T., Anderson N. L., Barnes J. M., Haigh E. A. P., & Fresco D. M. (2014). Using the GAD-Q-IV to identify generalized anxiety disorder in psychiatric treatment seeking and primary care medical samples [Article]. Journal of Anxiety Disorders, 28(1), 25–30. 10.1016/j.janxdis.2013.10.009 24334213

[31] Moulds M. L., Bisby M. A., Wild J., & Bryant R. A. (2020). Rumination in posttraumatic stress disorder: A systematic review. Clinical Psychology Review, 82, 101910. 10.1016/j.cpr.2020.101910 32971312

[32] Newman M. G., Llera S. J., Erickson T. M., Przeworski A., & Castonguay L. G. (2013). Worry and generalized anxiety disorder: A review and theoretical synthesis of research on nature, etiology, and treatment. Annual Review of Clinical Psychology, 9(1), 275–297. 10.1146/annurev-clinpsy-050212-185544 PMC4964851 23537486

[33] Newman M. G., Zuellig A. R., Kachin K. E., Constantino M. J., Przeworski A., Erickson T., & Cashman-McGrath L. (2002). Preliminary reliability and validity of the Generalized Anxiety Disorder Questionnaire-IV: A revised self-report diagnostic measure of generalized anxiety disorder. Behavior Therapy, 33(2), 215–233. 10.1016/S0005-7894(02)80026-0

[34] Nolen-Hoeksema S. (1991). Responses to depression and their effects on the duration of depressive episodes. Journal of Abnormal Psychology, 100(4), 569–582. 10.1037/0021-843X.100.4.569 1757671

[35] Nolen-Hoeksema S., & Morrow J. (1993). Effects of rumination and distraction on naturally occurring depressed mood. Cognition and Emotion, 7(6), 561–570. 10.1080/02699939308409206

[36] Nolen-Hoeksema S., Wisco B. E., & Lyubomirsky S. (2008). Rethinking rumination [Peer Reviewed]. Perspectives on Psychological Science, 3(5), 400–424. 10.1111/j.1745-6924.2008.00088.x 26158958

[37] Oathes D. J., Ray W. J., Yamasaki A. S., Borkovec T. D., Castonguay L. G., Newman M. G., & Nitschke J. (2008). Worry, generalized anxiety disorder, and emotion: Evidence from the EEG gamma band [Peer Reviewed]. Biological Psychology, 79(2), 165–170. 10.1016/j.biopsycho.2008.04.005 18499328 PMC2597009

[38] Ottaviani C., Thayer J. F., Verkuil B., Lonigro A., Medea B., Couyoumdjian A., & Brosschot J. F. (2016). Physiological concomitants of perseverative cognition: A systematic review and meta-analysis [doi:10.1037/bul0000036]. Psychological Bulletin, 142(3), 231–259. 10.1037/bul0000036 26689087

[39] Papageorgiou C., & Wells A. (Eds.). (2004). Depressive rumination: Nature, theory and treatment. Wiley Online Library. 10.1002/9780470713853.

[40] Psychology Software Tools inc. (2002). E-Prime 2.0. Pittsburgh, PA, USA.

[41] Robinson M. S., & Alloy L. B. (2003). Negative cognitive styles and stress-reactive rumination interact to predict depression: A prospective study. Cognitive Therapy and Research, 27(3), 275–291. 10.1023/A:1023914416469

[42] RStudio Team. (2023). RStudio: Integrated Development for R. In (Version 2023.12.0+369) RStudio, PBC, Boston, MA. http://www.rstudio.com/

[43] Sarisoy G., Pazvantoğlu O., Özturan D. D., Ay N. D., Yilman T., Mor S., Korkmaz I. Z., Kaçar Ö. F., & Gümüş K. (2014). Metacognitive beliefs in unipolar and bipolar depression: A comparative study. Nordic journal of psychiatry, 68(4), 275–281. 10.3109/08039488.2013.814710 23902127

[44] Segerstrom S. C., Tsao J. C. I., Alden L. E., & Craske M. G. (2000). Worry and rumination: Repetitive thought as a concomitant and predictor of negative mood. Cognitive Therapy and Research, 24(6), 671–688. 10.1023/A:1005587311498

[45] Silveira É. d. M. Jr, & Kauer-Sant’Anna M. (2015). Rumination in bipolar disorder: A systematic review. Brazilian Journal of Psychiatry, 37(3), 256–263. 10.1590/1516-4446-2014-1556 26176599

[46] Steer R. A., Ball R., & Ranieri W. F. (1999). Dimensions of the Beck Depression Inventory-II in clinically depressed outpatients. Journal of clinical psychology, 55(1), 117–128. 10.1002/(SICI)1097-4679(199901)55:1&lt;117::AID-JCLP12&gt;3.0.CO;2-A 10100838

[47] Szkodny L. E., & Newman M. G. (2019). Delineating characteristics of maladaptive repetitive thought: Development and preliminary validation of the Perseverative Cognitions Questionnaire. Assessment, 26(6), 1084–1104. 10.1177/1073191117698753 28355881 PMC6707362

[48] Tallis F., Davey G. C. L., & Bond A. (1994). The Worry Domains Questionnaire. In Davey G. C. L. & Tallis F. (Eds.), Worrying: Perspectives on theory, assessment and treatment. (pp. 285–297). John Wiley & Sons.

[49] Tallis F., Eysenck M. W., & Mathews A. (1992). A questionnaire for the measurement of nonpathological worry. Personality and Individual Differences, 13(2), 161–168. 10.1016/0191-8869(92)90038-Q

[50] Topper M., Molenaar D., Emmelkamp P. M., & Ehring T. (2014). Are rumination and worry two sides of the same coin? A structural equation modelling approach. Journal of Experimental Psychopathology, 5(3), 363–381. 10.5127/jep.038813

[51] Tull M. T., Hahn K. S., Evans S. D., Salters-Pedneault K., & Gratz K. L. (2011). Examining the role of emotional avoidance in the relationship between posttraumatic stress disorder symptom severity and worry. Cognitive Behaviour Therapy, 40(1), 5–14. 10.1080/16506073.2010.515187 21337211

[52] Watkins E., & Brown R. (2002). Rumination and executive function in depression: An experimental study. Journal of Neurology, Neurosurgery & Psychiatry, 72(3), 400–402. 10.1136/jnnp.72.3.400 11861707 PMC1737771

[53] Watkins E., Moulds M., & Mackintosh B. (2005). Comparisons between rumination and worry in a non-clinical population. Behaviour Research and Therapy, 43(12), 1577–1585. 10.1016/j.brat.2004.11.008 16239152

[54] Whitmer A. J., & Gotlib I. H. (2012). Switching and backward inhibition in major depressive disorder: the role of rumination. Journal of Abnormal Psychology, 121(3), 570. 10.1037/a0027474 22468767 PMC11877650

[55] Zainal N. H., & Newman M. G. (2018). Worry amplifies theory-of-mind reasoning for negatively valenced social stimuli in generalized anxiety disorder. Journal of Affective Disorders, 227, 824–833. 10.1016/j.jad.2017.11.084 29254067 PMC6707505

